# Determinants of emergency response responsibility perceptions in the local public health workforce after China’s health sector restructuring

**DOI:** 10.1186/s12913-015-1003-0

**Published:** 2015-08-21

**Authors:** Mingli Jiao, Ning Ning, Qunhong Wu, David H. Peters, Yanhua Hao, Ye Li, Xingang Wei, Zheng Kang

**Affiliations:** Department of Health Policy and Hospital Management, Health Management School of Harbin Medical University, 157 Baojian Road, Harbin City, Heilongjiang Province China; Department of Social Medicine, School of Public Health, Harbin Medical University, 157 Baojian Road, Harbin City, Heilongjiang Province China; Department of International Health, Johns Hopkins University Bloomberg School of Public Health, 615 N Wolfe St., Baltimore, MD 21205 USA; Health Bureau of Heilongjiang Province, Heilongjiang, Peoples Republic of China; Institute of Quantitative and Technical Economics, Chinese Academy of Social Science, 5 Jian guo men nei Road, Dongcheng District, Beijing, China

## Abstract

**Background:**

Local health departments are the backbone of public health emergency (PHE) response plans. The front line of emergency response preparedness is people. Role perceptions of individual staff members of a given organization strongly affect response probability and performance. Therefore, the aim of this study was to determine local public health employees’ perceptions of emergency response responsibilities, identify factors that influence their perception, and indicate the challenges and bottlenecks of PHE response in the Health Inspection Institution (HII) after its separation from China’s multiple Centers for Disease Control and Prevention (CDC).

**Methods:**

We used a stratified randomized sample survey to examine HII workers’ knowledge of their own duties concerning PHE response in 17 facilities in Heilongjiang, a province in northeastern China. Data were collected from May to July 2010 using a 9-item combined question inquiring about the workers’ statutory duties.

**Results:**

Of 348 administered surveys, 309 were returned for an overall response rate of 88.8 %. Overall, the correct recognition rate of PHE responsibilities was low. Some HII workers were confused about their responsibilities required by law, regulations, and emergency response plans. A quarter of all the respondents had the lowest knowledge for PHE responsibilities. Factors influencing their perceptions of responsibilities were department, work experience in a CDC, and PHE response experience.

**Conclusions:**

To improve preparedness for a PHE, efforts are needed to train, support, and monitor the workers’ knowledge and competencies in PHEs as part of an organizational change; the worker’s knowledge of their responsibilities should be measured and used as an indicator of preparedness for a PHE, and training should be undertaken where there are deficiencies. Management should also encourage workers in the departments of food hygiene/school health surveillance to be more involved in PHE preparedness and response issues.

## Background

Recent years have witnessed a number of microbial threats that have greatly jeopardized public health in many areas of the world. One of the most publicized cases is severe acute respiratory syndrome (SARS), whose outbreak and rapid spread in more than 25 countries in early 2003 claimed numerous lives and caused tremendous economic losses. It is predicted that new pathogens—originating naturally or from bioterrorism—will continue to emerge and cause new public health emergencies (PHEs) [1–4]. Response to PHEs should be regarded as a global issue and particular attention should be paid to developing countries that have relatively fewer resources to deal with PHEs [1].

The outbreak of SARS exposed a fundamental shortcoming not only in China’s public health system but also in China’s limited ability to detect and respond to emergencies in a timely and effective manner [1, 5]. Realizing the weakness of its public health systems, China is undertaking extensive efforts to improve its public health system by establishing and strengthening its “one mechanism and three systems” approach: the PHE response mechanism, disease control and prevention, medical treatment, and the health inspection and health law enforcement system. As one of the newest responsibilities assigned to public health agencies after SARS, PHE response is at the forefront of public attention and involves numerous players; the major providers are in healthcare sectors such as health bureaus, hospitals, multiple Centers for Disease control and Prevention (CDCs), and Health Inspection Institutions (HIIs).

Local health departments are considered the backbone of public health response plans for any and all PHEs [6]. The PHE response during the Ebola epidemic that occurred in West Africa in 2014 reiterated this position. The HII has been established as a new branch of China’s public health system after separation from the CDCs under China’s health sector reform and subsequent organizational transformation in 2010. Increasing HII preparedness for disasters—especially local preparedness—is a significant concern of public health planners because several studies suggest that the initial response to a PHE would generally begin at the local level [7–9].

The front line of emergency response preparedness is people [10]. Role perceptions of individual staff members of a given organization will factor strongly into response probability and performance [11, 12]. Job responsibility awareness has always been imperative for people to perform tasks effectively [13]. We define job responsibility as a state in which the individual perceives an obligation for a situation or event [14].

The shortage of job knowledge and job responsibility might decrease job performance, because job knowledge predicts job performance [15]. Daniel and his colleagues at the Johns Hopkins Center for Public Health Preparedness identified specific barriers to adopting an emergency response culture in local health departments. These barriers include public health employees’ fear for their personal and family safety in emergencies, coupled with a lack of insight into how valuable they are to crisis response efforts [16, 17]. Differences in perceptions of responsibilities in PHE preparedness across administrative levels is another barrier, because different public health information, programs, and distribution channels may be required to increase preparedness among different subgroups. Thus, understanding each individual’s role in response to a PHE is considered key for competent response [18].

However, with the separation from the CDC and the addition of PHE response as a new function came many challenges for the HIIs in terms of implementing the work: the current health supervision system model is not uniform and personnel status is not clear, among other issues. The most critical challenge is the absence of clearly defined duties and responsibilities. The assignment of responsibility and the response procedures have not been specified in detail [19]. Several overlapping roles and responsibilities between the CDC system and the HII system have hindered their interagency cooperation. The CDC possesses techniques for public health surveillance, while the HIIs have the authority to conduct surveillance. In practice, some staff complain that they always feel embarrassed because they cannot decide how, where, when, or how frequently to conduct surveillance. Many projects have to be delayed owing to poor cooperation [3, 14].

Previous studies have shown that during extreme scenarios, a majority of healthcare workers may be unable or unwilling to report to duty [16]. This may be true even for local health departments. Little is known about administrative differences in HII preparedness—especially after the separation of the HII from the CDC [18]. Few studies, however, have focused on the preparedness and competent response of local HIIs in China for policy makers. One reason might be that HII only recently became a separate agency from the CDCs.

As the major provider in dealing with PHEs after the 2003 SARS crisis, do HII workers clearly know their duties related to the PHE? What factors influence their perception? What are the challenges and bottleneck problems? The solution to these essential questions will be effective in generating a competent PHE response. Therefore, the aim of this study was to assess local public health employees’ perceptions of emergency response responsibilities after China’s health sector restructuring, and to uncover the variables that affect these outcomes, thus providing much needed evidence for health departments’ training efforts. In this study, we demonstrate that the CDCs and HIIs have not yet formed a closely coordinated relationship after the sector restructuring. Further, HII workers still do not clearly understand their PHE responsibilities and this lack of understanding could also occur during reforms of health sectors in other countries.

## Methods

### Study design

The present study used a quantitative approach, analyzing HII workers’ perception of responsibilities associated with a PHE and the determinants to indirectly assess the development and status of HII emergency preparedness capacity at the individual level in Heilongjiang. We developed the study objectives and questionnaire in collaboration with representatives of the Heilongjiang Public Health System (Health Bureau, CDC, HII, and public hospital) and piloted it in Harbin, the capital city of Heilongjiang province.

### Ethics approval

The study protocol was reviewed and approved by the Research Ethics Committee of Harbin Medical University. All participants indicated their willingness to take part, both verbally and in consent forms signed before the commencement of interviews.

### Data collection

This study was undertaken in Heilongjiang province, northeast China. Heilongjiang has 13 cities. The questionnaire survey was conducted with the stratified-cluster sampling method. Considering the geographical and jurisdictional diversity, we first divided Heilongjiang’s 13 cities into three subgroups according to 4 indicators: (a) gross domestic product per person, (b) population mortality in 2000, (c) life expectancy in 2000, and (d) childhood mortality in 2000 [20, 21]. Then, three cities—Harbin, Mudanjiang, and Yichun (and their counties or districts)—were selected from these three subgroups on the basis of the other two indicators, which was the number of PHEs per 10,000 population (≥0.75, national average level) and the coverage rate of health supervision (≥80 %, national average level) [22]. These three cities have a total of 48 agencies for HIIs. After providing a detailed description of the purpose of the survey, 17 facilities expressed an interest to participate in the survey (1 provincial, 3 municipal, and 13 county and district HIIs).

Subsequently, the researchers visited these 17 agencies to conduct the face-to-face interviews. All the staff at each agency participated in the survey except those on leave. Finally, we obtained 309 completed questionnaires (response rate: 88.8 %) from health inspectors (with inspector licenses granted by China’s Ministry of Health); data collection occurred during May to July 2010.

### Dependent variables

Dependent variables indicated the outcome of staff perceptions of their job responsibilities associated with a PHE. The response duties of staff from healthcare sectors (health bureaus, hospitals, CDCs, and HIIs) have been summarized in 4 clauses [1, 5], which are further classified into 9 categories (Table 1). We assessed the perception of job responsibility among HII practitioners using a 9-item combined question inquiring about these statutory duties (SD; items 1, 3, 5, 7, and 8): “Among these 9 items, which are the SDs of your health inspection institution? (1) Under the direction of local health administrations, conduct an inspection of the PHE response measures of hospitals, (2) Conduct epidemiologic investigations, (3) Conduct an inspection of the PHE response measures of CDCs, (4) Public information release, (5) Supervise the PHE response process; (6) Organize/conduct a mass vaccination campaign, (7) Conduct an inspection of the enforcement of laws and regulations related to PHE, (8) Assist the local health administration to carry out investigations into irregularities of the PHE response, (9) Supervise the PHE response process at health administrations.” Allowed responses were “yes,” “no,” or “don’t know.” Item 2, 4, 6, 9 are the responsibilities of other health sectors: CDCs or health bureaus; the correct answer for these items was “no.”

**Table 1:** Duties of healthcare sectors during a public health emergency outbreak

Provisions Source	Duties	The responsible sector
National public health emergency response plan	(1) Under the direction of local health administrations, conduct inspection regarding the PHE response measures of hospitals	HII
National health sector health emergency management specification	(2) Conduct epidemiologic investigation	CDC
Public health emergencies and infectious diseases surveillance information report management approach	(3) Conduct inspection over the PHE response measures of CDCs	HII
	(4) Public information release	Health bureaus
	(5) Conduct supervision over the process of PHE response	HII
	(6) Organize/conduct a mass vaccination campaign	CDC
	(7) Conduct inspection over the enforcement of laws and regulations related to PHE	HII
Several provisions of the health supervision system	(8) Assist the local health administration to carry investigations into irregularities of PHE response	HII
	(9) Conduct supervision over the process of PHE response of health administrations	Health bureaus

### Independent variables

We selected independent variables based on discussions with representatives of the Heilongjiang Public Health System. The variables included gender, age, educational level and background, job content, work experience in a CDC, PHE training/drills experience, PHE response experience, and administrative levels: provincial, municipal, or county/district HIIs.

PHE training/drills experience was defined as a positive response to the following question: “Have you participated in any PHE training, exercise, or drill?” Allowed responses were “yes,” “no,” or “don’t know.” Work experience in a CDC was defined as a positive response to the following question: “Have you ever worked in a CDC?” Allowed responses were “yes,” “no,” or “don’t know.” PHE response experience was defined as a positive response to: “How many times have you participated in PHE responses?” Allowed responses were “0 = never participated” or the number of times they had participated.

Finally, the variable job content was defined as whether the respondents engaged in the following 6 items: (1) medical institutions inspection, infectious disease supervision, and mother and baby care surveillance; (2) food hygiene and school health surveillance; (3) health inspection of public facilities, cosmetics, and drinking water; (4) occupational health and radioactive surveillance; (5) health law enforcement and supervision; (6) health inspection and enforcement information management. Allowed responses were “yes” or “no.”

### Statistical analysis

Data were analyzed using SAS version 9.1. We analyzed the responses of 309 participants after excluding 39 participants with responses of “don’t know” or who refused to answer any of the questions. Descriptive statistics were calculated to describe the demographic characteristics. Differences among the provincial, municipal, county/district groups were determined using chi-square and two-tailed *t*-tests for categorical and continuous variables, respectively. Fisher’s exact test was used if subgroups included less than 10 subjects.

The staff’s correct recognition of SDs and the proportion of ways to recognize their own job responsibilities related to PHE response were detected using chi square tests (*χ*^2^). Univariate analysis was performed to characterize the sample, followed by bivariate analysis to determine the relationship between dependent and independent variables.

Finally, multiple variable logistic regression analyses were performed with the correct recognition of PHE responsibilities as the dependent variable. We derived a dichotomous indicator for the dependent variables: coded “1” if the responses to all 5 SDs were “yes,” and coded “0” if otherwise. All regression models generated adjusted odds ratios (AORs) and 95 % confidence intervals (CIs) that measured the independent relationship of each covariate to the outcome variables, after we adjusted for confounding by the other covariates. Statistical significance was defined as P < 0.05.

## Results

### Characteristics of the sample

Characteristics of the sample by provincial, municipal, and county/district levels are shown in Table 2. Of the total sample (n = 309), most participants (74.76 %) were 30 to 50 years of age; most of the respondents (45.95 %) reported having a college or graduate degree, but with notable differences between the groups: 74.14 % of the provincial group, 61.11 % of the municipal group, and 27.33 % of the district/county group (P < 0.0001). Overall, approximately a quarter of the respondents’ job content involved food hygiene/school health surveillance (26.86 %) and health inspection for public facilities/cosmetics/drinking water (24.27 %).

**Table 2:** Characteristics of study participants (n = 309)

Characteristics		N (%)	Provincial HII (%)	Municipal HII (%)	County/District HII (%)	*P*
Gender						
Men		171 (55.34)	31 (53.45)	43 (52.22)	93 (57.76)	0.6634
Women		138 (44.66)	27 (46.55)	43 (47.78)	68 (42.24)	
Age						
20 ~ 39		126 (40.78)	17 (29.31)	35 (38.89)	74 (45.96)	0.0965
40 ~ 49		126 (40.78)	27 (46.55)	34 (37.78)	65 (40.37)	
50~		57 (18.44)	14 (24.14)	21 (23.33)	22 (13.66)	
Education level						
High school or less		49 (15.86)	3 (5.17)	9 (10.00)	37 (22.98)	<.0001
Community College		118 (38.19)	12 (20.69)	26 (28.89)	80 (49.69)	
College/graduate		142 (45.95)	43 (74.14)	55 (61.11)	44 (27.33)	
Education Background						
Public health		49 (15.86)	32 (55.17)	49 (54.44)	81 (50.31)	0.7030
Clinical Medicine		118 (38.19)	12 (20.69)	25 (27.78)	41 (25.47)	
Others		142 (45.95)	14 (24.14)	16 (17.78)	39 (24.22)	
Department						
Medical Institutions/Disease/Mother & Care	Infectious Baby	55 (17.80)	12 (20.69)	23 (25.56)	20 (12.42)	0.1466
Food Hygiene/School Health Surveillance		83 (26.86)	17 (29.31)	19 (21.11)	47 (29.19)	
Public facilities/cosmetics/drinking water		75 (24.27)	12 (20.69)	20 (22.22)	43 (26.71)	
Occupational/radiation		46 (14.88)	8 (13.79)	15 (16.67)	23 (14.29)	
Administrative Enforcement		32 (10.36)	3 (5.17)	11 (12.22)	18 (11.18)	
Information Management/other		18 (5.83)	6 (10.34)	2 (2.22)	10 (6.21)	
Work Experience in CDC						
Yes		166 (53.72)	32 (55.17)	29 (32.22)	105 (65.22)	<.0001
No		143 (46.28)	26 (44.83)	61 (67.78)	56 (34.78)	
PHE Training/Drills						
Yes		187 (50.49)	18 (31.03)	53 (58.89)	116 (72.05)	<.0001
No		122 (49.51)	40 (68.97)	37 (41.11)	45 (27.95)	
Public health emergency Response Experience						
Yes		156 (50.49)	26 (44.83)	51 (56.67)	79 (49.07)	0.3250
No		153 (49.51)	32 (55.17)	39 (43.33)	82 (50.93)	
Total		309				

In the county/district HIIs, more people had undergone PHE training or drills, and more people had work experience in a CDC (all P < 0.001; Table 2). Overall, 50.49 % of respondents underwent PHE training/drills, with the county/district group having the highest reported frequency (72.05 %), followed by the municipal group (58.89 %) and the provincial group (31.03 %). When asked whether they had work experience in a CDC, over half of the respondents (53.72 %) reported they had worked in a CDC before the separation of the HII from a CDC; the county/district group had the highest reported proportion of participants with work experience in a CDC (65.22 %) compared to the provincial group (55.17 %) and municipal group (32.22 %). Furthermore, 50.49 % of the respondents reported that they had participated in some kind of PHE response; there were no significant group differences.

### Correct recognition of statutory duties

Overall, 54.37 % (n = 309) of respondents picked out 5 SDs from the 9-item combined question. Among the three groups, the municipal group had the highest correct recognition rate (57.78 %), followed by the county/district group (55.28 %) and provincial group (46.55 %); the differences were significant (P <0.05). Different correct recognitions of SDs by level are shown in Table 3. SD 3 (supervise the PHE response process) had the highest correct recognition rate (94.50 %) and SD 2 (conduct an inspection of the PHE response measures of the CDC) had the lowest recognition rate (69.90 %).

**Table 3:** Staff’s correct recognition of Statutory Duties (SD) by administrative level (n = 309)

Questions/Answers	N (%)	Provincial HII (%)	Municipal HII (%)	County/District HII (%)	*χ* ^2^	*P*
SD 1						
Yes	261 (84.47)	46 (79.31)	82 (91.11)	133 (82.61)	Fisher	0.0094
No	26 (8.41)	3 (5.17)	3 (3.33)	20 (12.42)		
Unknown	22 (7.12)	9 (15.52)	5 (5.56)	8 (4.97)		
SD 2						
Yes	216 (69.90)	34 (58.62)	61 (67.78)	121 (75.16)	7.8527	0.1042
No	59 (19.10)	13 (22.41)	18 (20.00)	28 (17.39)		
Unknown	34 (11.00)	11 (18.97)	11 (12.22)	12 (7.45)		
SD 3						
Yes	292 (94.50)	51 (87.93)	84 (93.33)	157 (97.52)	Fisher	0.0030
No	8 (2.59)	1 (1.72)	5 (5.56)	2 (1.24)		
Unknown	9 (2.91)	6 (10.34)	1 (1.11)	2 (1.24)		
SD 4						
Yes	280 (90.61)	48 (82.76)	86 (95.56)	146 (90.68)	Fisher	0.0978
No	15 (4.86)	4 (6.90)	2 (2.22)	9 (5.59)		
Unknown	14 (4.53)	6 (10.34)	2 (2.22)	6 (3.73)		
SD 5						
Yes	247 (79.94)	36 (62.07)	78 (86.67)	133 (82.61)		0.0002
No	28 (9.06)	8 (13.79)	2 (2.22)	18 (11.18)		
Unknown	34 (11.00)	14 (24.14)	10 (11.11)	10 (6.21)		
Total	309					

*SD 1: “Under the direction of the local health administrations, conduct inspection of the PHE response measures of hospitals”; SD 2: “Conduct inspection of the PHE response measures of the CDC”; SD 3: “Conduct supervision of the process of PHE response”; SD 4: “Conduct inspection of the enforcement of laws and regulations related to PHE”; SD 5: “Assist local health administration to conduct investigations into irregularities of PHE responses”

There were notable differences among the three levels in the correct recognition rate of SDs 1, 3, and 5; the responses showed a similar trend: the correct recognition rates of the municipal and county/district groups were better than those of the provincial group.

### Recognition of job responsibilities related to PHE

Table 4 shows the observed frequency of responses to the question, “How do you recognize your job responsibilities related to a public health emergency?” Overall, the job description was the main method through which the workers recognized their PHE responsibilities: it had the highest percentage of “yes” responses (58.90 %), followed by the emergency plan (57.93 %), and the official written assignment (54.05 %). However, the assignment had the highest “yes” responses in the provincial group: more than half of the respondents (53.45 %) reported that they knew their responsibilities through the assignment; in the municipal and county/district groups, the predominant ways to recognize one’s responsibilities were the emergency plan (75.56 %) and job description (58.39 %).

**Table 4:** Proportion of ways to know their own job responsibilities related to Public Health Emergency response, by administrative level

Questions/Answers	N (%)	Provincial HII (%)	Municipal HII (%)	County/District HII (%)	*P*
Emergency Plan					
Yes	179 (57.93)	24 (41.38)	68 (75.56)	87 (54.04)	<.0001
No	130 (42.07)	34 (58.62)	22 (24.44)	74 (45.96)	
Assignment					
Yes	167 (54.05)	31 (53.45)	47 (52.22)	89 (55.28)	0.8925
No	142 (45.95)	27 (46.55)	43 (47.78)	72 (44.72)	
Job Description					
Yes	182 (58.90)	30 (51.72)	58 (64.44)	94 (58.39)	0.3021
No	127 (41.10)	28 (48.28)	32 (35.56)	67 (41.61)	
Self learning					
Yes	35 (11.33)	13 (22.41)	7 (7.78)	15 (9.32)	0.0118
No	274 (88.67)	45 (77.59)	83 (92.22)	146 (90.68)	
Other					
Yes	11 (3.56)	2 (3.45)	1 (1.11)	8 (4.97)	0.0272
No	298 (96.44)	56 (96.55)	89 (98.89)	153 (95.03)	
Total	309				

There was no group difference with respect to the assignment (P = 0.8925) or job description (P = 0.3021). Conversely, there was a group difference with respect to the emergency plan and self-learning: these had the greatest effect on the municipal group (75.56 % vs. 41.38 % on the provincial group and 54.04 % on the county/district group, P < 0.0001), followed by the provincial group (22.41 % vs. 9.32 % on the county/district group and 7.78 % on the municipal group, P <0.05) and county/district group (4.97 %, P <0.05).

### Influencing factors associated with job responsibility awareness

Multivariate logistic regression revealed that job content, work experience in a CDC, and PHE response experience factors were independently related to an increased correct recognition rate of job responsibilities associated with PHE responses (Table 5). Respondents whose job content involved supervision of medical institutions/infectious disease/mother & baby care (AOR = 5.031, 95 % CI = 2.235–11.323), public facilities/cosmetics/drinking water (AOR = 2.255, 95 % CI = 1.129–4.505), occupational/radiation (AOR = 3.450, 95 % CI = 1.542–7.716), or administrative enforcement (AOR = 4.413, 95 % CI = 1.711–11.378) were more likely than those with a job content involving food hygiene/school health surveillance or information management/other to exhibit correct knowledge of their PHE responsibilities. Respondents with work experience in a CDC were more likely to know their PHE responsibilities better than persons without CDC work experience (AOR = 1.886, 95 % CI = 1.094–3.249); respondents who reported PHE response experience exhibited knowledge of their PHE responsibilities more clearly than people without PHE response experience (AOR = 2.089, 95 % CI = 1.174–3.716).

**Table 5:** Results of full multivariate logistic models of Awareness of Job Responsibility of Public Health Emergency in HIIs^a^

Variable^b^	*SE*	*P*	Adjusted odds ratio ( 95 % CI^c^ )
Gender	0.2699	0.0918	1.576 (0.929 ~ 2.675)
Age	0.1873	0.2615	0.810 (0.561 ~ 1.170)
Education			
Community College	0.3843	0.3292	1.455 (0.685 ~ 3.090)
College/graduate degree	0.4437	0.5958	1.265 (0.530 ~ 3.020)
Education background	0.3191	0.0858	1.730 (0.926 ~ 3.233)
Clinical Medicine			
Others	0.3167	0.9306	1.028 (0.553 ~ 1.912)
Job contents			
Supervision of Medical Institutions/Infectious Disease/Mother & Baby Care	0.4139	<.0001	5.031 (2.235 ~ 11.323)
Public facilities/cosmetics/drinking water	0.3530	0.0212	2.255 (1.129 ~ 4.505)
Occupational/radiation	0.4107	0.0026	3.450 (1.542 ~ 7.716)
Administrative Enforcement	0.4833	0.0021	4.413 (1.711 ~ 11.378)
Information Management/other	0.5919	0.1575	2.309 (0.724 ~ 7.366)
Work Experience in CDC			
Yes	0.2776	0.0223	1.886 (1.094 ~ 3.249)
PHE Training/Drills			
Yes	0.3060	0.1571	1.542 (0.846 ~ 2.809)
PHE Response Experience			
Yes	0.2938	0.0122	2.089 (1.174 ~ 3.716)

Note. ^a^HII = Health Inspection Institution. ^b^Reference groups are male, public health education background, high school or less graduation, Food Hygiene/School Health Surveillance job content, reported no Work Experience in a CDC, had no PHE Training/Drills experience, and no PHE Response Experience^c^ CI = confidence interval

## Discussion

### HII workers do not understand their PHE responsibilities

Although a decade and a year has passed since the 2003 SARS outbreak and subsequent separation of the HII from the CDC with the Chinese public health system rebuilding and restructuring, HII staff members still do not clearly understand their job responsibilities associated with PHE response. Our data showed that almost 55 % of respondents identified the 5 SDs from the 9-item combined question. Among them were respondents who chose more responsibilities from the 9-item question (items 2, 4, 7, 9 are the responsibilities of other health sectors: CDCs or health bureaus) as SDs. Thus, only 10.03 % answered the question accurately; namely, after the separation of the HII from the CDC, only one-tenth of respondents not only knew what their functional role requirements were in a PHE response as required by regulations, laws, and China’s National Public Health Emergency Planning, but they also knew what were not their duties (some of them are duties of the CDC). Furthermore, the item “Conduct an inspection of the PHE response measures of the CDC” had the lowest correct recognition rate (69.90 %, Table 3) among the 5 SDs. This result is consistent with those of one study showing that the relationship between the CDC and HII had not yet been coordinated since the Chinese public health system was divided into the multiple CDCs and the HII. The CDCs possess techniques for public health surveillance, while the HII possesses the authority to conduct surveillance [19]. However, many HII workers still confuse the HII’s responsibilities with the CDC’s.

Combined with the actual situation in China, in theory, when a PHE occurs, according to the needs of the event itself, the Ministry of Health will set up an emergency squad composed of health professionals from the CDCs, HIIs, and other emergency agencies [20]. For example, the HII and cosmetics and drinking water departments may be temporarily called to participate in emergency work and would be required to fulfill the emergency duties planned. However, in reality, if a large-scale PHE does occur in China, special department personnel are not enough. These situations, for example, a large-scale drinking water contamination, call for increased manpower; whenever possible, staff from the food sector will be transferred to offer temporary support. When the emergency squad needs a health supervisor, the department’s most experienced employees may take precedence, but this selection of department staff may not strictly depend on the nature of the epidemic. Therefore, the duties prescribed in the plan are macroregulations for an overall HII. In China, the PHE responsibilities do not involve correspondence with other departments [21]. Therefore, staff from each healthcare sector do not have specific responsibilities, which results in overlapping responsibilities between the HII and CDC and other healthcare sectors as well as between the departments in the HII. This in turn causes delays in PHE response and reduced response efficiency when the HII has to conduct an inspection of the PHE response measures of the CDC or assist the local health administration to investigate irregularities in PHE responses. The existence of poor communication between these departments was observed in the present survey conducted: SD 2 and SD 5 were associated with the poorest responses.

In comparison, health sectors in Canada and the United States have clearly outlined roles, with no cross-functional associations and relatively independent work [22]. In Australia, the existing health supervision and management systems were adopted from the British. Here, health supervision and other regulations have implementation details, and are founded by strong operational regulations, system, and hierarchy. We can learn and implement much from the Federal Emergency Management Agency in the United States: increase tasks not agencies, but feel free to adjust the goals, focus, and department in accordance with the actual situation to improve emergency response capabilities in China [23–26].

### Educational level does not explain the difference in PHE responsibility perception

We initially thought that the provincial group would have had the highest correct recognition rate because it had the highest educational level of the three groups [27, 28]. However, in the present study, the respondents’ educational level was higher than that of the general educational status of China’s HIIs: 45.95 % of participants reported college or above education levels compared to the 27.1 % reported across the nation [29–31]. Our results also demonstrated that educational levels do not explain the difference between PHE responsibility recognition rates in these groups. Overall, the PHE responsibility recognition in municipal and county/district groups was better than the provincial group’s—this was unexpected because the provincial group had higher educational levels than the other 2 groups, and, because the provincial HII was located in the capital city, the workers were theoretically more likely to access PHE response information and be exposed to high-end technology [32–35].

Also important is the notable difference in PHE training/drills; lower-level HIIs reported a higher frequency of training/drills. That might explain the higher PHE recognition in the lower HIIs. That fact also highlights the necessity of organizing multisector response drills. The other reason may be that lower-level HIIs have fewer workers and have to play multifaceted roles. There are about 10 workers in each county/district HII and their jobs will vary with needs; thus, they had some general knowledge in different areas.

### Low PHE responsibility-recognition levels among food hygiene/school health surveillance workers

As previously suggested by Benatar (1997, 1998) [36, 37], the duty to care must be placed in a wider context to include considerations that transcend individual obligations. As Bensimon’s study illustrates, the broader institutional and societal context must be taken into account.

This finding suggests that interventions need to target workers in the departments of food hygiene/school health surveillance. At the same time, since the job content of over a quarter of the respondents in our study was food hygiene/school health surveillance, such interventions might be the most effective way to enhance the levels of PHE responsibility recognition. Job content, having work experience in a CDC, and having experience with previous PHE response factors influence the correct recognition rate of job responsibilities associated with a PHE.

More than a quarter of the total respondents were food hygiene/school health surveillance workers, but their PHE responsibility recognition level was the worst. Increasing the food hygiene/school health surveillance workers’ recognition of PHE responsibilities is significant for improving PHE preparedness and response, especially since China’s Ministry of Health announced that the top two major PHE causes are food poisoning, with the high-risk location being schools—especially primary and middle schools in rural areas [38].

Most food hygiene/school health surveillance workers used to be food hygiene supervisors before HII became an independent agency of the public health system. At that time, their job responsibility focused on inspection of food hygiene, and they adhered to outdated working practices: the job content did not require inspection of hospitals and CDCs. The workers were, therefore, not familiar with the new requirements such as supervising PHE response measures of hospitals regarding the PHE response process or assisting local health administrations investigate irregularities in the PHE response. If left unresolved, this situation might pose a weak link in China’s PHE response chain.

### Factors influencing PHE responsibility recognition

Finally, multivariate logistic regressions revealed a strong association between responses involving correct recognition rates for the 5 SDs and job contents, work experience in a CDC, and having PHE experience. Workers in the department of food hygiene/school health surveillance and information management/other had decreased recognition of their correct PHE responsibilities. They had the lowest correct recognition rate of the 5 SDs. Having work experience in a CDC and having personal experience in disaster response, however, were associated with increased recognition of PHE responsibilities. This result was consistent with that in a study wherein personal experience with disasters was associated with increased adoption of protective behaviors [39].

This may require innovative training, severe punishment, and organizational development methods, such as those in place in Canada and Australia, to enhance preparedness skills and create a culture of organizational readiness among public health workers through a process of “syntonic” organizational change [40]. In the syntonic model, organizational change is a nonthreatening, natural growth process that is more likely to be embraced by employees than resisted; key factors in syntonic change include anticipatory guidance and experiential learning [40]. Although the assignment of responsibility and response procedures have not been specified in detail, overall, the job description (58.90 %) was the most powerful way to help HII workers to recognize their duties associated with a PHE response, followed by the emergency plan (57.93 %) and the assignment (54.05 %). The emergency plan is the fundamental structure in municipal HIIs; three-fourth of municipal respondents reported that they recognized their PHE responsibilities through the emergency plan, compared to 4 of 10 provincial HII workers and half of the county/district workers. The assignment also played a critical role, and there was no difference between the 3 groups; over 50 % of the workers perceived their responsibilities through the assignment.

This study has several limitations. First, data were collected using self-assessment tools, and therefore, the general level of emergency preparedness may be overestimated. Second, the study’s time and resource restrictions confined the investigation to 17 agencies in one province, the data for which may not be generalizable to other geographic areas. However, the findings of this research could be used in emergency response procedures in the future. Through the examination of HII workers’ knowledge of their own duties, it may be possible to improve the efficiency of the workforce in the HII during PHE response.

## Conclusions

The circumstances surrounding the SARS crisis made it both possible and desirable to explicitly address the question of duty to care. Our study demonstrates that after the sector restructuring in China, the CDCs and HIIs have not yet formed a closely coordinated relationship. Our study reveals that HII workers still do not clearly understand their PHE responsibilities and that this lack of understanding could also occur during reforms of health sectors in other countries.

The outcome of our study also highlights the complexities of organizational restructuring, which may decrease PHE preparedness and response capacity. A lack of standard and basic requirements for PHE response—such as general tasks, functions, and responsibilities in routine job descriptions—will ultimately hinder efficient behaviors during the PHE response process. To improve preparedness for a PHE, perceptions of responsibilities should be used as an additional indicator of preparedness. Furthermore, from the perspective of globalization significance, China is facing the same problems, including delayed initiation and slow development of the PHE response, such as many other developing countries. These results may be useful to guide preparedness interventions in developing countries and future research.

## Abbreviations

CDC: Centers for Disease Control
HII: Health Inspection Institution
PHE: Public Health Emergency
